# Efficient Interleaved Multi-Band Outer Volume Suppression for Highly Accelerated Simultaneous Multi-Slice Imaging of the Heart

**DOI:** 10.3390/bioengineering13030286

**Published:** 2026-02-28

**Authors:** Ayda Arami, Omer Burak Demirel, Toygan Kilic, Steen Moeller, Yidong Zhao, Yi Zhang, Qian Tao, Hildo J. Lamb, Mehmet Akçakaya, Sebastian Weingärtner

**Affiliations:** 1 Department of Imaging Physics, Delft University of Technology, 2628 CJ Delft, The Netherlands; a.arami@tudelft.nl (A.A.); y.zhao-8@tudelft.nl (Y.Z.); y.zhang-43@tudelft.nl (Y.Z.); q.tao@tudelft.nl (Q.T.); 2 Department of Radiology, Leiden University Medical Center, 2333 ZG Leiden, The Netherlands; h.j.lamb@lumc.nl; 3 Philips North America, Rochester, MN 55902, USA; burak.demirel@philips.com; 4 Department of Electrical and Computer Engineering, University of Minnesota, Minneapolis, MN 55455, USA; kili0040@umn.edu (T.K.); akcakaya@umn.edu (M.A.); 5 Center for Magnetic Resonance Research, University of Minnesota, Minneapolis, MN 55455, USA; moell018@umn.edu

**Keywords:** simultaneous multi-slice imaging, outer volume suppression, cardiac MRI

## Abstract

In this work, we aimed to develop and evaluate multi-band outer volume suppression pulses for increased acceleration rates in simultaneous multi-slice accelerated cardiac MRI. MB-OVS pulses were constructed from a multi-band combination of two slab-selective saturation pulses and tested for various pulse shapes using Bloch simulation and phantom experiment. The MB-OVS pulses were interleaved between imaging pulses to ensure homogeneous suppression throughout the cardiac cycle/imaging window in vivo. Simultaneous multi-slice (SMS) CINE and first-pass myocardial perfusion scans with and without the proposed MB-OVS pulses were compared in terms of residual artifacts at high acceleration rates. Among the tested pulses, both Bloch simulation and phantom experiments showed that amplitude-optimized sinc pulses provided the best trade-off in suppression efficiency, the required $B_{1}^{+}$, SAR, and slab profile. CINE imaging with 5-fold SMS-OVS acceleration significantly outperformed imaging without MB-OVS, maintaining leakage-free image quality, even when adding 2-fold in-plane acceleration. SMS-OVS also enabled perfusion imaging in 9 slices with 1.7 × 1.7 mm^2^ resolution, achieving a 16-fold spatial-only acceleration while ensuring accurate contrast dynamics without leakage artifacts. Interleaved MB-OVS modules enabled thorough leakage artifact suppression in cardiac SMS-accelerated CINE and perfusion imaging, particularly at high acceleration rates. The proposed approach may be promising for unlocking further acceleration potential of SMS in cardiac imaging.

## 1. Introduction

Cardiac Magnetic Resonance Imaging (MRI) is increasingly being used in the clinical work-up of ischemic and non-ischemic heart diseases [1], and is adopted in a growing number of disease-specific guidelines [2]. Cardiac CINE imaging is the clinical gold standard for the measurement of cardiac function [3], and first-pass myocardial perfusion MRI is widely accepted for the assessment of Coronary Artery Disease (CAD) [4]. However, long scan times, limited resolution, and coverage in cardiac exams remain major challenges for more widespread clinical adoption [5,6].

Image acceleration based on the use of multiple receiver coils is widely used in clinical MRI in the form of parallel imaging [7]. This approach routinely enables acceleration rates up to 2-to-3-fold [8,9,10,11]. Parallel imaging is commonly used to reduce the number of required Phase-Encoding (PE) lines in multi-slice 2D imaging to shorten the end-diastolic acquisition window. Simultaneous Multi-Slice (SMS) imaging, on the other hand, enables more efficient coverage and, thus, substantial savings in scan time [12]. Furthermore, SMS incurs no Signal-to-Noise Ratio (SNR) reduction related to acceleration rate, with the only noise penalty stemming from the coil geometry [13]. While SMS has facilitated the next leap in scan time acceleration in neuro MRI [14,15,16,17,18], its use in cardiac imaging has been limited [19,20,21,22,23,24,25]. The spatial overlap between unfavorable coil sensitivity profiles and hyperintense signals from the back and the chest often leads to leakage artifacts in cardiac SMS imaging [20]. This makes the application of SMS in the heart challenging, particularly with high acceleration rates and when applied in combination with parallel imaging for in-plane acceleration. In first-pass perfusion, previous studies have explored SMS acceleration rates of up to 3- combined with 5.33-fold acceleration in plane [11], or SMS-only acceleration up to 4 [21]. CINE imaging has been performed with up to 4-fold SMS factor without in-plane acceleration [26].

Slice-leakage artifacts can be mitigated using reconstruction approaches that exploit differences in coil sensitivity across slice locations [12]. Linear reconstruction approaches, such as split-slice Generalized Autocalibrating Partially Parallel Acquisitions (GRAPPA) and iTerative Self-consistent Parallel Imaging Reconstruction (SPIRiT), are widely used [27,28]. However, they can amplify noise in a coil-geometry-dependent manner and propagate slice leakage linearly, where the residual signal from one slice contaminates another [27]. Consequently, strong extra-cardiac signals can produce pronounced artifacts within the heart, particularly at high SMS acceleration factors. Non-linear reconstruction methods incorporate regularization or deep learning to further reduce slice leakage while limiting noise amplification. Recently, a non-linear approach called ROCK (ReadOut Concatenated K-space with)-SPIRiT has been proposed, in which they combine the leakage-blocking properties of RO (Read-Out)-SENSE (SENSitivity Encoding)-GRAPPA [29] with SPIRiT-based coil self-consistency constraints; with additional regularization, they further suppress noise [30]. This hybrid approach targets both limitations simultaneously. Although it improves CINE imaging relative to GRAPPA or SPIRiT alone [30], extension to first-pass perfusion remains challenging, as time-varying contrast during bolus passage can introduce residual aliasing and temporal blurring, including blurred Left Ventricular (LV) uptake. Physics-guided deep learning [31] may help address these issues. In this regard, Signal-Intensity-Informed Multi-coil (SIIM) encoding within Physics-Guided Deep Learning (PG-DL) networks has demonstrated improved reconstruction quality for highly accelerated perfusion CMR [32].

Alternatively, slice leakage artifacts can be addressed at the acquisition stage by suppressing the outer volume signal prior to reconstruction. Outer Volume Suppression (OVS) has previously been proposed to reduce artifacts originating from peripheral body parts in various MRI applications [33,34,35]. For cardiac imaging, different OVS schemes have been explored. Traditionally, slab-selective saturation pulses were used to mitigate back or chest signals [35,36]. More recently, 2D spatially selective excitation pulses have been proposed to suppress extra-cardiac tissue in both in-plane dimensions [37,38]. A combination of selective and non-selective flip-down/-up pulses in T_2_ preparations has also been suggested for OVS imaging in coronary MR Angiography (MRA) [39,40]. However, due to signal recovery during the imaging windows, OVS modules need to be interleaved with the imaging pulses for optimal and homogeneous suppression. In cardiac MRI, this leads to a trade-off between suppression efficiency, Specific Absorption Rate (SAR), pulse duration, and the disruption of the magnetization steady-state.

In this work, we sought to develop and validate a Multi-Band Outer Volume Suppression (MB-OVS) module for highly accelerated cardiac SMS imaging. We proposed that the interleaved use of MB-OVS modules enables homogeneous suppression of extra-cardiac signals and improves SMS accelerated cardiac imaging. To this end a MB-OVS, comprising two saturation bands that are played simultaneously for time-efficient signal suppression, is implemented. The module was integrated in an interleaved way in the CINE and perfusion sequences. Various RF pulses were explored to achieve favorable trade-offs between leakage artifact suppression, SAR, slice profile characteristics, and duration. Bloch simulations were used to study the slice profile, the required $B_{1}^{+}$, sensitivity to off-resonance, and $B_{1}^{+}$ scale. Next, phantom experiments were performed to validate the simulation results, steady-state disruption, and measure SAR. Finally, the acceleration potential in vivo was evaluated using SMS CINE and first-pass perfusion imaging, combined with nonlinear reconstruction to mitigate slice leakage.

## 2. Materials and Methods

### 2.1. MB-OVS Design and Sequence

**Figure 1:**
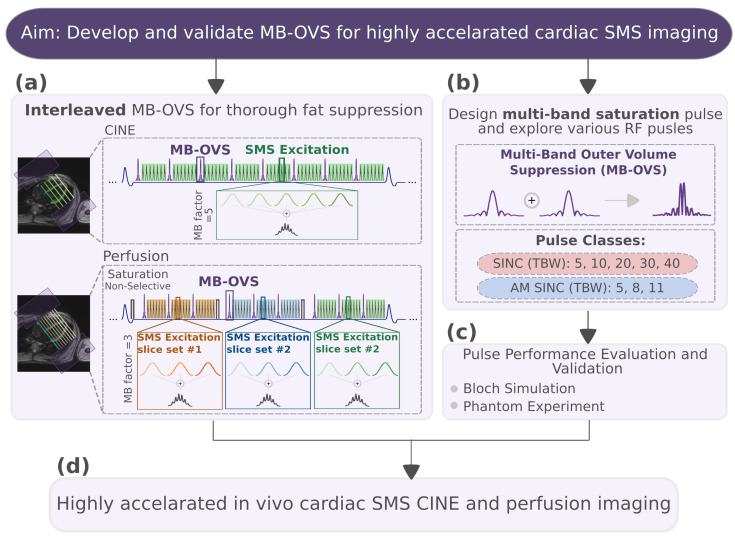
Study design. (**a**) Schematic pulse sequence for SMS-accelerated CINE and perfusion imaging with interleaved MB-OVS. The sums of five (CINE) and three (perfusion) sinc-pulses at different center frequencies are depicted for excitation pulses in spGRE imaging. By interleaving these excitation pulses, OVS is achieved throughout the acquisition window. Example orientations of short-axis slices alongside the suppression bands in the anterior–posterior phase-encoding direction are indicated in the 4-chamber images on the left. (**b**) MB-OVS pulses are obtained as the sum of two single-band pulses to simultaneously suppress the unwanted signals in the two parallel slabs depicted with purple boxes in panel (**a**). Two pulse types were investigated for MB-OVS: SINC and AM SINC pulses. (**c**) Pulse performance was evaluated through Bloch simulation and phantom experiments to identify the optimal design in terms of slice profile, $B_{1}^{+}$, SAR, and duration. (**d**) The selected pulse was subsequently validated in highly accelerated SMS CINE and perfusion imaging.

A multi-band combination of two slab-selective saturation pulses was used in the MB-OVS module to simultaneously suppress chest and back signals from parallel slabs along the PE dimension. The multi-band excitation enabled minimal pulse duration and, thus, minimal disruption of the magnetization evolution during the imaging window. The schematics of a CINE and a perfusion sequence using the proposed MB-OVS pulses are shown in Figure 1. For CINE, the MB-OVS pulses were interspersed throughout the k-space readout. For perfusion, the MB-OVS modules were played before the image readout and interleaved with the excitation pulses. This way, homogeneous suppression can be achieved throughout the acquisition. All imaging was performed on a 3T scanner (Magnetom Prisma, Siemens Healthineers, Erlangen, Germany) using a 30-channel receiver coil.

### 2.2. Bloch Simulation

MB-OVS performance was characterized through Bloch simulations. The simulations evaluated residual signal suppression, pulse characteristics, and robustness to field inhomogeneities. Specifically, the following were performed:

- Residual signal analysis across a range of $T_{1}$ values using interleaved MB-OVS in the spGRE CINE sequence;
- Slice profile comparison for SINC and AM SINC pulses, including slice-selective gradient strength and $B_{1}^{+}$ requirements;
- $B_{1}^{+}$ scale and off-resonance effects on the chosen pulse to assess robustness in the presence of system imperfections.

### 2.3. Phantom Imaging

Phantom imaging was performed using AMplitude-optimized (AM SINC) and conventional sinc (SINC) pulses for slab-selective saturation in the MB-OVS module. A 12 cm-diameter homogeneous water-filled sphere was placed between two 8 cm-long fat bottles to coarsely resemble chest and back tissues around the heart. CINE imaging was performed using a simulated electrocardiogram (ECG) signal at 60 beats per minute (bpm) with 25 retrospectively gated cardiac phases. Signal suppression efficiency was calculated by comparing it to standard CINE imaging without MB-OVS modules. AM SINC with Time-Bandwidth product (TBW) of 5, 8, and 11 were compared with SINC with TBW of 5, 10, 20, 30, and 40. An additional reference image was acquired, where the RF power of the MB-OVS pulse was set to zero to investigate the effect of disrupting the steady state in isolation. The signal suppression efficiency was quantified as the residual signal in the stop-bands using manually drawn Regions-Of-Interest (ROIs), normalized to the signal in the baseline images without MB-OVS.

The following imaging parameters were used for spoiled GRE (spGRE) CINE imaging in phantom: TR/TE/FA = 5.6/2.15 ms/12°, bandwidth = 770 Hz/Px, FOV = 320 × 320 mm^2^, resolution = 1.8 × 1.8 mm^2^, slice-thickness = 6 mm, temporal resolution = 40 ms. For the MB-OVS module, a 150 mm-thick slab selective 90° saturation for each side and RF peak shift of 15%, was followed by 200 μs gradient spoiling. Thus, the total duration of 4.6 s was achieved for the MB-OVS module. The MB-OVS preparation was interleaved between every 8 imaging pulses.

### 2.4. In Vivo MB-OVS Validation

This study was approved by the institutional review board (No. 1509M77881), and written informed consent was obtained before each examination.

#### 2.4.1. CINE Imaging

MB-OVS accelerated CINE imaging was performed in 3 healthy subjects and 2 patients (4 Men, 1 Woman, 55±15). CINE imaging was performed following perfusion imaging, as recommended for scan time-efficient cardiac protocols [41]. Sequence parameters for in vivo CINE imaging were TR/TE/FA = 4.3/2.1 ms/12°, FOV = 320 × 320 mm^2^, resolution = 1.7 × 1.7 mm^2^, slice-thickness = 8 mm, temporal resolution = 41–55 ms, breath-hold duration = 15–17 s, and SMS-factor = 5. To further accelerate, the dataset was retrospectively undersampled with a two-fold in-plane acceleration while keeping the Auto Calibration Signal (ACS) = 24 reference lines. Following the phantom results, a 3.8-millisecond AM SINC 8 was used in the MB-OVS module and interleaved every 8 imaging pulses. CINE images were reconstructed offline in MATLAB R2023a (MathWorks, Natick, MA, USA) using ROCK-SPIRiT with Locally Low-Rank (LLR) regularization as previously proposed [30].

Single-band CINE images with and without MB-OVS were compared to visualize suppression efficiency. The effect of MB-OVS on volumetric measurements obtained from accelerated CINE images was assessed in comparison with the single-band reference. The LV was automatically segmented for each cardiac phase separately, using a nnU-Net framework [42] with uncertainty estimation [43,44]. The difference in volumetry was assessed as the Root Mean Square (RMS) difference of the volumetric curve compared to the single-band reference. Paired Student’s *t*-tests were used to compare RMS values with and without MB-OVS, for both with and without additional in-plane acceleration. Statistical significance was set at p<0.05.

#### 2.4.2. Myocardial Perfusion Imaging

Myocardial perfusion imaging was performed in 6 healthy subjects (5 Men, 1 Woman, 39±19 y/o). Imaging was performed for three cardiac phases, each following a non-selective saturation and a short saturation delay (Figure 1). A composite saturation pulse comprising four individual pulses was used [45]. An injection of 0.05 mmol/kg gadobutrol (Gadovist, Bayer HealthCare Pharmaceuticals, Berlin, Germany) was administered at 4 mL/s, followed by a 10 mL saline flush.

Imaging sequence parameters were TR/TE/FA = 2.9/1.7 ms/12°, FOV = 360 × 360 mm^2^, resolution = 1.7 × 1.7 mm^2^, slice-thickness = 8 mm, temporal resolution = 125 ms, saturation time = 150 ms, SMS-factor = 3, in-plane acceleration = 4, and partial-Fourier = 6/8 (overall 16-fold acceleration). The same MB-OVS module parameters from CINE Imaging were used and interleaved every 9 imaging pulses. PD-DL with SIIM was used for the reconstruction of myocardial perfusion images with self-supervised training [46,47] as previously proposed [32]. PD-DL with SIIM encoding and Self-Supervised learning via Data Undersampling (SSDU) training was performed using PyTorch 2.0.0 and processed on a workstation with an Intel E5-2640V3 CPU and an NVIDIA Tesla V100 GPU.

The accelerated MB-OVS perfusion imaging data was assessed in terms of contrast uptake. For each slice, all dynamics were registered in a template-free group-wise manner using principal component analysis [48,49]. The signal uptake was then extracted using ROIs manually drawn into the mid-ventricular septum and the left-ventricular blood pool. Low-pass filtering using a moving average was applied to the uptake curves [50].

## 3. Results

### 3.1. Bloch Simulation

**Figure 2:**
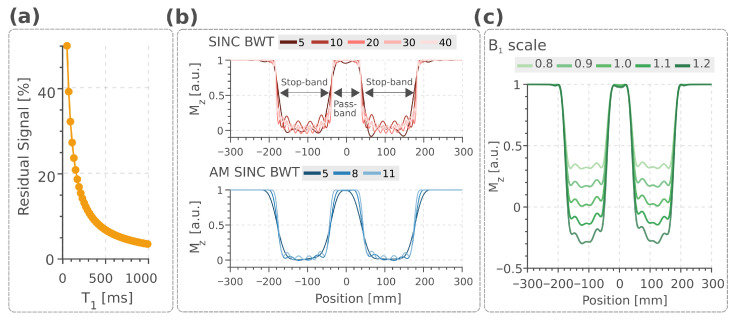
(**a**) Simulated residual signal for interleaved MB-OVS in spGRE CINE acquisition across $T_{1}$ values. At $T_{1}$ = 200 ms, the residual signal reached 16.06%. (**b**) Slice profile comparison between SINC and AM SINC class of pulses for the MB-OVS. For both pulse types, pass-band-to-stop-band transition sharpness improved with increasing TBW, while SINC pulses showed stronger sidelobe artifacts. SINC AM provided an optimal compromise among duration, required $B_{1}^{+}$, and selectivity. Sensitivity of the AM SINC 8 to $B_{1}$ inhomogeneity was shown in (**c**).

Figure 2a demonstrates 10–15% residual signal variation across the $T_{1}$ range of 250–350 ms (approximating subcutaneous fat at 3T [51]), with higher values at shorter $T_{1}$. The increased residual signal reflected greater longitudinal magnetization recovery occurring between successive OVS modules for tissues with shorter $T_{1}$. Figure 2b compares the slice profiles of SINC and AM SINC pulses across different TBW. Increasing TBW sharpened the stop-band-to-pass-band transition for both pulse categories. This improvement came at different costs for each pulse type. At higher TBW, SINC pulses exhibited side lobes positioned closer to the stop-band with increased amplitude. While for AM SINC pulses, the required $B_{1}^{+}$ increased with higher TBW (Table 1). Gradient strengths were fixed at 407 μT/m for all SINC pulses and 203, 326, and 448 μT/m for AM SINC pulses with TBW of 5, 8, and 11, respectively (Table 1). AM SINC 8 offered an optimal compromise between pulse duration, $B_{1}^{+}$ requirements, and slice profile homogeneity.

### 3.2. Phantom Imaging

**Figure 3:**
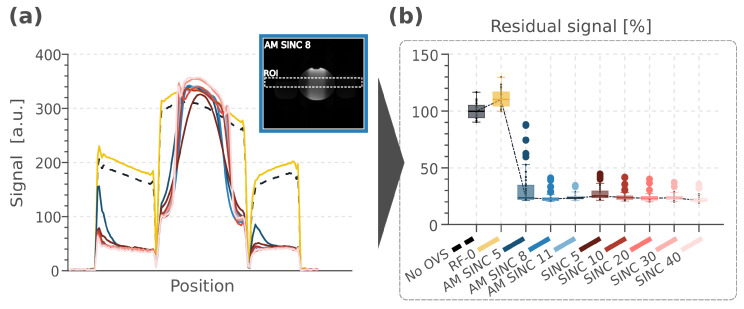
(**a**) Slab profiles of the various RF pulses within the ROI (white dashed box) are shown as signal-intensity plots along the phase-encoding direction. Within the pass-band, the imaging signal remains largely unchanged, while it is suppressed in the stop-band; the phantom image on the corner illustrates MB-OVS performance for the AM SINC 8, which was selected for use in the remainder of the study. (**b**) The residual signal in the stop-band relative to the signal in the baseline images without MB-OVS, using the different MB-OVS pulses.

Pulse characteristics and suppression performance in the phantom experiments are shown in Figure 3 and Table 1. The stop-band signal on both sides of the phantom was successfully suppressed in all MB-OVS pulses compared to the acquisition without MB-OVS. The different RF pulses achieved similar levels of signal suppression (residual signal: 20–30%), albeit at different SAR levels and pulse durations. SINC 40 demonstrated the lowest residual signals but required pulse durations exceeding 10 ms. In contrast, AM SINC pulses achieved comparable residual signal levels at much shorter durations. Among these, AM SINC 8 showed the least residual signal (23.72%) while maintaining a relatively low SAR (1.26 W/kg). Furthermore, as illustrated in Figure 3a, and in agreement with Bloch simulations (Figure 2), AM SINC pulses maintained a smooth slab profile, whereas SINC pulses exhibited side lobes that distorted the water signal in the pass-band. Figure 3a also showed that AM SINC 5 exhibited the most pronounced slice profile shift due to its weaker gradient strength. This resulted in more severe slice profile distortion. Overall, AM SINC 8 was chosen as a trade-off between pulse duration, SAR, suppression efficiency, and slab profile and was utilized in all in vivo studies. AM SINC 8 pulses were applied every 8 pulses in the CINE sequence. This yielded a total SAR increase of 1.17 and 1.19 W/kg relative to conditions without MB-OVS (No OVS) and with the MB-OVS module at zero RF power (RF-0), respectively.

### 3.3. In Vivo SMS Imaging

#### 3.3.1. CINE Imaging

**Figure 4:**
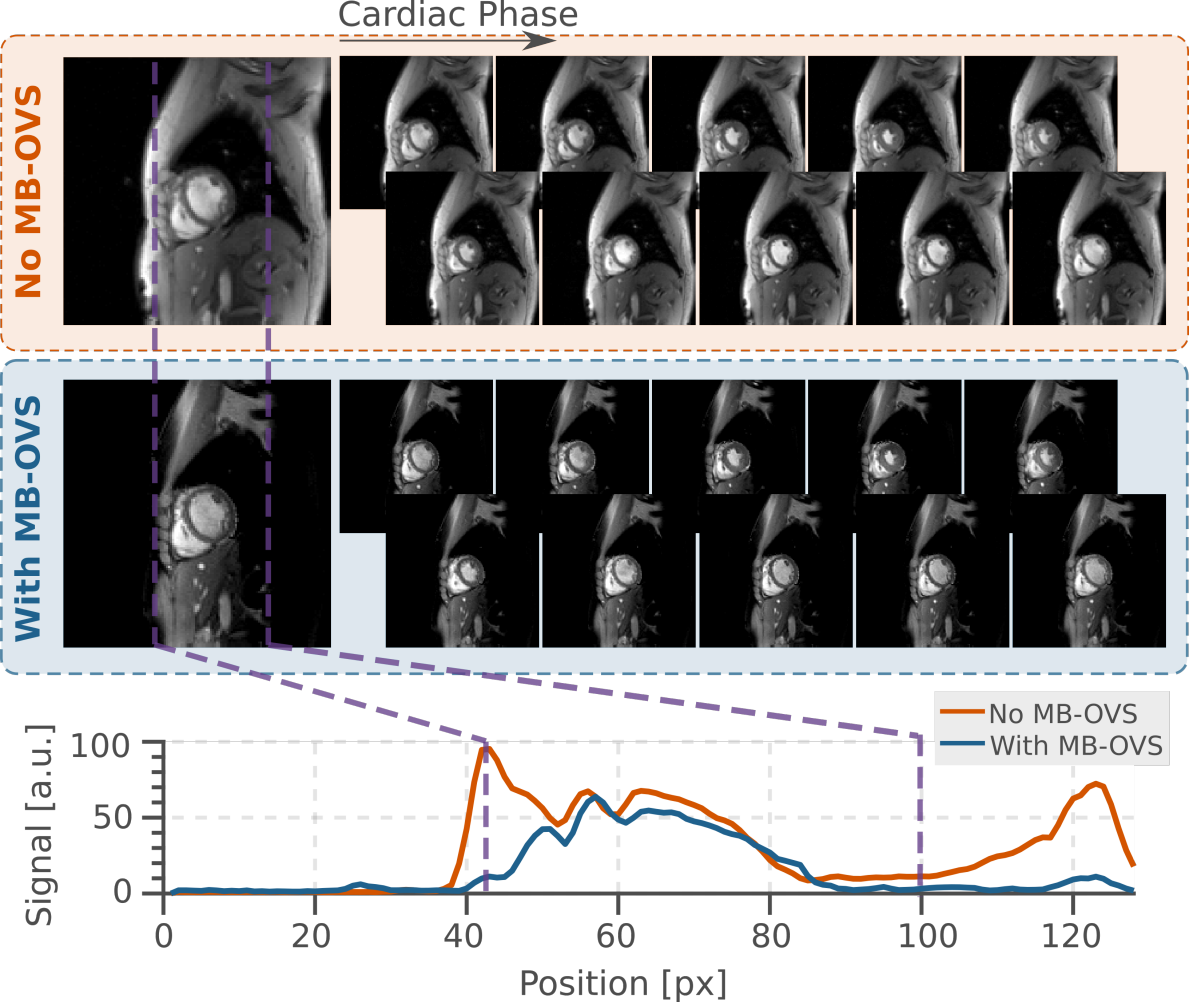
Illustration of proposed MB-OVS in CINE imaging with single-band excitation in the baseline images and as signal intensity plots along the phase encoding direction (anterior–posterior). The purple dashed lines mark the boundary between the pass- and stop-bands. Good signal intensity reduction, especially near the back and the chest fat, is homogeneously maintained throughout all phases of the cardiac cycle.

Figure 4 shows the effects of MB-OVS pulses using AM SINC 8 in CINE imaging without SMS acceleration. In visual assessment, the chest and back signals were thoroughly suppressed. The difference in signal quality in the pass-band was negligible compared with the images without MB-OVS.

**Figure 5:**
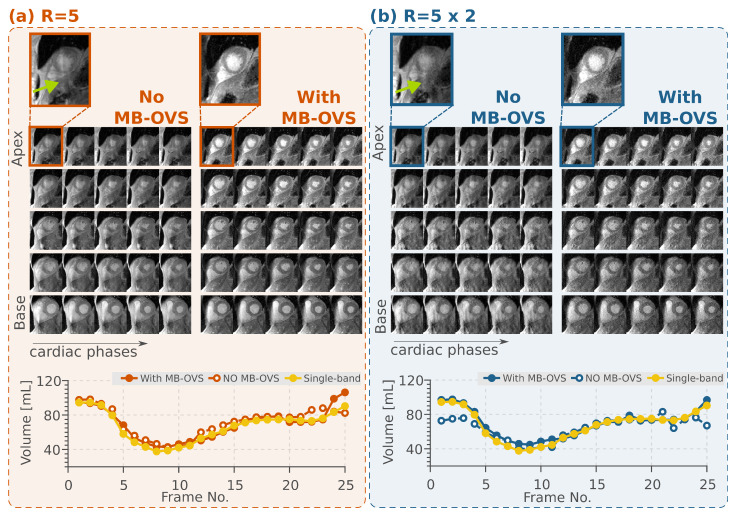
(**a**) Five-fold SMS accelerated CINE images with and without MB-OVS. Conventional SMS imaging without MB-OVS shows severe leakage artifacts in all 5 slices, compromising the depictionof the blood–myocardium interface. When MB-OVS is utilized, no residual artifacts are observed with a clear blood–myocardial delineation at the left ventricle; the zoomed-in panel (top) highlights the slice leakage artifact indicated by the green arrow. (**b**) CINE imaging with an additional 2-fold in-plane acceleration with and without MB-OVS. Although noise amplification is observed at 10-fold acceleration, images with MB-OVS show no leakage artifacts, while the image quality further deteriorates without MB-OVS. Similar to panel (**a**), the zoomed-in view of the frame on top highlights the slice leakage artifact. Left ventricular volume quantification, for 5- and 10-fold accelerated CINE imaging, with and without MB-OVS in comparison to the single-band reference, are shown at the bottom of panels (**a**) and (**b**), respectively.

Figure 5a shows results obtained with five-fold SMS accelerated images using the proposed MB-OVS in CINE imaging in one healthy volunteer. SMS without MB-OVS showed substantial signal leakage onto the heart that hinders the depiction of the blood myocardial interface in visual assessment. MB-OVS pulses restored high image quality without visually apparent residual leakage artifacts throughout all cardiac phases. Implementation of MB-OVS every 8 imaging pulses resulted in an increase in SAR from 0.41 ± 0.08 W/kg to 1.64 ± 0.09 W/kg for the spGRE CINE sequence.

Further acceleration by an additional retrospective in-plane sampling of two (total 10-fold) is shown in Figure 5b with and without using MB-OVS pulses. At this acceleration rate, the proposed MB-OVS approach still resulted in leakage-free images, although with a visually apparent increase in noise levels. The volume quantification throughout the cardiac cycle for the same subject is shown in Figure 5.

Over all subjects, the pre-trained nnUNet segmentation network yielded comparable differences in volumetry with respect to the single-band reference for all accelerations with and without MB-OVS, despite visually apparent artifacts when no MB-OVS was used; without in-plane acceleration: with MB-OVS 20.64±12.25 mL, without MB-OVS 19.69±22.50 mL (p=0.92), and with additional in-plane acceleration: with MB-OVS 21.89±14.72 mL, without MB-OVS 28.03±34.24 mL (p=0.64). No statistically significant differences were found between MB-OVS and without MB-OVS conditions in either acceleration scenario (p>0.05 for both comparisons). However, in the cases with the strongest leakage artifacts, as observed with an additional 2-fold in-plane acceleration and without MB-OVS, segmentations were noticeably misaligned with the myocardium (Supplementary Materials Video S1). Stable segmentation is restored in this case when MB-OVS is employed (Supporting Information Video S2).

#### 3.3.2. Myocardial Perfusion Imaging

**Figure 6:**
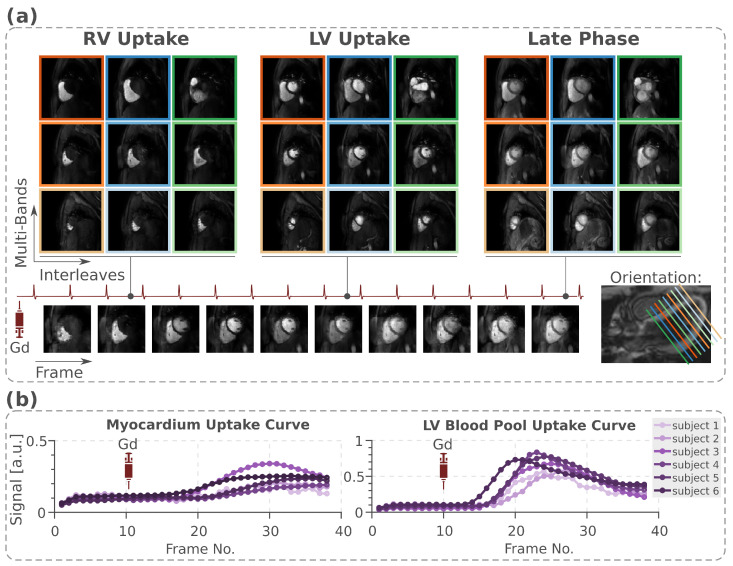
(**a**) Accelerated perfusion imaging with 3-fold SMS × 4-fold in-plane acceleration × 6/8 partial for a total 16-fold acceleration. A total number of 9 slices with 3 imaging sets, each has 3-fold SMS acceleration, is acquired per heartbeat, providing whole heart coverage. No visually apparent residual leakage or blurring is observed across the 9 slices. (**b**) The uptake curve is measured for the LV blood pool and myocardium across all subjects.

Figure 6 depicts the first-pass myocardial perfusion images with 3-fold SMS acceleration, 4-fold in-plane acceleration, and 6/8 partial Fourier (with a total of 16-fold spatial-only acceleration) using MB-OVS pulses and reconstructed using SIIM. Despite low baseline SNR in the saturation-recovery sequence, blood pools were clearly delineated from the myocardium, and no residual inter-slice leakage or blurring is apparent. With the sequence and MB-OVS parameters used in this study, perfusion imaging can be performed for subjects with heart rates up to 90 bpm. The mean heart rate of the study cohort was 74.84 ± 19.81 bpm.

Uptake curves for the LV blood pool and the myocardium are shown for all subjects in Figure 6b. Due to the spatial-only acceleration, MB-OVS faithfully reflected contrast dynamics throughout the acquisition.

## 4. Discussion

In this study, an efficient MB-OVS imaging module for use in highly SMS-accelerated cardiac imaging was evaluated. Various RF pulses were investigated to yield optimal suppression at minimal pulse duration and SAR. In vivo images with 10-fold accelerated CINE and 16-fold accelerated perfusion imaging showed that the thorough suppression of the back and chest signal facilitates leakage-free reconstructions with SMS rates up to 5. Homogeneous signal suppression was achieved throughout the cardiac cycles in both CINE and perfusion imaging by using MB-OVS interleaved throughout the acquisition. Phantom and in vivo experiments revealed excellent image quality with high temporal and spatial resolution.

The use of multi-band excitation severely exacerbates the peak $B_{1}^{+}$ requirement and increases the SAR burden. Conventional SINC pulses concentrate RF energy in the central lobe, necessitating a higher peak $B_{1}^{+}$ compared with AM SINC pulses. In this study, peak shifting [52] was applied to alleviate the $B_{1}^{+}$ requirements at the trade-off against increased pulse duration. However, in interleaved OVS applications, pulse duration is an important trade-off, as longer pulses increasingly perturb steady-state magnetization and prolong acquisition windows. Therefore, AM-SINC 8 was chosen in this study as it demonstrated a favorable balance between slice profiles, peak $B_{1}^{+}$, SAR, and duration in phantom experiments.

OVS bands are typically positioned at the periphery, far from the isocenter. These areas are often characterized by strong $B_{0}$ inhomogeneities. Thus, resilience against off-resonance effects is paramount for robust stop-band delineation in OVS. All SINC pulses were played with a constant excitation bandwidth and thus, comparable resilience to off-resonance. To this end, the duration of the conventional SINC pulses was chosen proportionally to their TBW. Bloch simulations and phantom measurements showed that conventional SINC pulses produce significant side lobes that encroach on the pass-band. This substantially lowered the effectiveness of the outer volume suppression and can lead to artifacts stemming from the residual signals. AM-SINC pulses, on the other hand, were studied with different effective bandwidths, but with fixed duration. Thus, increasing TBW led to a higher slab-selection gradient strength, which yielded a sharper stop-band-to-pass-band transition. Phantom experiments confirmed that this increases robustness to off-resonance, due to smaller off-resonance-induced stop-band shifts. However, AM-SINC pulses with higher TBW required higher peak $B_{1}^{+}$ and increased SAR.

Shinnar–Le Roux (SLR) pulses, based on Finite Impulse Response (FIR) filter design, offer reduced required $B_{1}^{+}$ while preserving higher spatial selectivity [53,54]. When combined with higher-order phase modulation, SLR pulses distribute energy across multiple lobes instead of a single central lobe, thereby reducing peak $B_{1}^{+}$ requirements. However, bandwidth remains approximately linearly coupled with duration [54]. For further improved slice profiles in OVS without excessive TBW [54], adiabatic pulses can be used. Adiabatic pulses decouple duration and bandwidth while providing resilience to $B_{1}$ inhomogeneity [55]. Interleaved multi-band adiabatic implementations require further investigation, which remain areas for future work.

Different suppression schemes have previously been proposed for the suppression of unwanted signals in the FOV in cardiac imaging. For example, 2D pulses were previously used for signal suppression [37,56], enabling up to 5-fold accelerated perfusion MRI [37]; however, the pulse duration and SAR burden of 2D excitation hamper its use in the interleaved application. Additionally, in these studies [37,38] 2D signal suppression was required to avoid artifacts stemming from extra-cardiac signals when using a spiral trajectory. With Cartesian trajectories, on the other hand, slice leakage only occurs along the PE dimension. Thus, shorter 1D pulses may be preferred. Other works [39,40] developed an OVS-T_2_prep enabling up to 6-fold [40] accelerated MRA by suppressing surrounding tissue signals and enhancing blood-tissue contrast simultaneously. However, this approach is only suitable for applications that require T_2_ preparations. Thus, in this work, a short and low-SAR 1D OVS pulse design was proposed. For that purpose, a multi-band combination of two slab-selective saturation pulses was utilized to achieve saturation bands for suppression of chest and back signals.

Inner Volume Imaging (IVI) is an alternative to the OVS method that restricts the area in which the signal is excited and/or refocused. For spin echo imaging, IVI can be achieved by using orthogonal 1D-selective 90° excitation and 180° refocusing pulses [57]. Previous studies demonstrated the feasibility of IVI with spin echo imaging for myocardial $T_{2}$ mapping, reducing scan time from 26 s to 16 s without aliasing artifacts. However, compromised $T_{2}$ quantification was observed due to stimulated echo contributions [58]. Inner volume excitation can alternatively be achieved by replacing the conventional 1D slice-selective pulse with a 2D spatially selective RF pulse using either echo-planar [59] or spiral [60] readout trajectories. This has previously enabled the use of IVI in gradient-echo sequences [59,60]. However, the lengthy pulse duration of 2D pulses increases echo times, thereby compromising image quality and constraining the achievable excitation resolution. Unlike IVI, OVS typically serves as magnetization preparation, allowing flexible integration with different imaging sequences without compromising the echo or repetition times. The combination of IVI and OVS preparation pulse, however, can be explored to further enhance suppression performance. In select scenarios, this may enable higher acceleration potential or improved spatial resolution, particularly in the presence of dominant signals outside the region of interest.

Recently, a novel approach called region-optimized virtual coils [61] was proposed as another alternative to OVS pulses. This method employs beamforming based on the receiver coil sensitivities to suppress signals from unwanted regions. The region-optimized virtual coils approach was shown to facilitate CINE imaging with 2-fold SMS acceleration and 4-fold in-plane, for a total acceleration rate of 8 [62]. However, this technique is heavily dependent on the geometric characteristics of the receiver array, posing additional restrictions to those required for SMS imaging. This may hinder its applicability with standard clinical receive hardware. Nonetheless, as highlighted by the authors [61], the approach is greatly complementary with pulse-sequence-based OVS, and the combination of both warrants further investigation for facilitating even higher acceleration in cardiac SMS.

In this study, images were reconstructed using ROCK-SPIRiT [30] and PD-DL with SIIM [32] encoding for CINE and first-pass perfusion, respectively. ROCK-SPIRiT with low-rank regularization outperforms conventional leakage-reducing SMS reconstruction techniques in mitigating leakage and g-factor effects [30]. Thus, it facilitated visually artifact-free image quality at 10-fold acceleration. However, its application to perfusion imaging revealed limitations, particularly in the form of blurring during the LV uptake curve, as previously shown [32]. PD-DL incorporating SIIM encoding has demonstrated efficacy in mitigating aliasing artifacts and noise level reduction at higher acceleration [32]. In combination with the deep-learning reconstruction, the proposed MB-OVS module enabled a 16-fold spatial-only acceleration with a faithful representation of the temporal dynamics. A growing number of DL-based reconstructions have been proposed for accelerated cardiac MR in SMS reconstructions [31]. Due to the reduced extra cardiac fat, the proposed MB-OVS module is expected to contribute to reducing leakage in those reconstructions. Thus, a combination of the proposed MB-OVS module with other cutting-edge DL reconstructions warrants investigation for further scan time acceleration.

In CINE imaging, results demonstrated a clear distinction in leakage artifacts between images with and without MB-OVS, with artifacts worsening under an additional 2-fold in-plane acceleration. Despite these visual differences, volumetric measurements showed no significant differences in RMS values of volumetric measurement across all subjects in all accelerations. This can be attributed to the robustness of the ML segmentation algorithm [43,44], which provided realistic contour dimensions despite the visual corruption of the blood–myocardium interface. However, a case study in the Supplementary Materials highlighted the results with severe leakage artifacts from an additional 2-fold in-plane acceleration without MB-OVS, which led to significant myocardial segmentation errors. Implementing MB-OVS, in this case, restored stable segmentation, illustrating its potential to mitigate artifacts and improve segmentation accuracy at higher acceleration rates.

Incorporating MB-OVS into CINE and perfusion spGRE maintained low SAR in this study. The increase in SAR was primarily driven by the number of applied OVS pulses. Given the higher baseline SAR of bSSFP compared with spGRE, however, the use of interleaved MB-OVS may lead to high SAR values. This potentially necessitates further trade-offs in terms of TR or flip-angle. Thus, integration in clinical bSSFP protocols warrants further investigation.

This study has several limitations. Imaging was performed on a limited number of healthy subjects and only a small number of patients. No heart rates above 90 bpm were encountered in the present study cohort. Thus, application of the perfusion sequence in a single cardiac cycle was not impeded. However, higher heart rates and shorter R-R intervals require compromises among spatial resolution, slice coverage, and the number of MB-OVS modules in the interleaved implementation to shorten acquisition duration. Fewer MB-OVS modules may lead to inhomogeneous signal suppression from subcutaneous fat. This can be particularly critical in obese people, where leakage artifacts are exacerbated. For CINE imaging, retrospective gating used in this work helped compensate for heart rate variability during acquisition. However, higher heart rates would limit the number of cardiac phases that can be acquired. This reduces temporal resolution and the precision of cardiac dynamic assessment. Thus, future parameter optimization is needed to accommodate higher and variable heart rates. Additionally, results may differ for patients with larger body habitus, varying subcutaneous fat, and different heart rate ranges. This highlights the need for validation across a more diverse and larger population. Non-adiabatic pulses were utilized with the advantage of low SAR requirements to enable repeated and interleaved applications even at 3T. However, non-adiabatic pulses are sensitive to B_1_ inhomogeneities and off-resonance artifacts. Future studies are warranted to assess the robustness of the proposed technique in the presence of major field inhomogeneities, such as those caused by implants. Lastly, while bSSFP is widely used for CINE imaging, its strong sensitivity to disruptions of the steady state complicates the integration of interleaved OVS. For this reason, CINE acquisitions in the present study were performed using spGRE. For bSSFP CINE, interleaved OVS can potentially be achieved using +/−alpha/2 pulses before and after the OVS module to store the magnetization along the longitudinal axis during the MB-OVS module. Investigating such an implementation is left for future research.

## 5. Conclusions

In this study, the proposed MB-OVS module enables up to 10-fold and 16-fold spatial acceleration in SMS CINE and first-pass perfusion imaging, respectively. This work suggests that MB-OVS can provide a practical advance for accelerated cardiac SMS imaging by efficiently suppressing extra-cardiac signals. Good suppression was observed for subcutaneous fat, which is a particularly strong source of slice leakage artifacts in cardiac SMS imaging. Owing to the interleaved application of the module, the proposed approach ensures thorough magnetization suppression across cardiac phases. Multi-band excitation helped optimize the temporal efficiency of the module, minimizing disruption to the transient or steady-state magnetization. By suppressing extra-cardiac signals prior to reconstruction, MB-OVS alleviates potential fold-over or slice leakage artifacts that are often observed with parallel imaging and SMS acceleration. Combining MB-OVS with nonlinear SMS reconstruction further reduces residual aliasing and noise amplification.

The proposed interleaved MB-OVS works well with spGRE sequences, but integrating interleaved MB-OVS into bSSFP sequences requires further investigation. The short temporal duration of the MB-OVS module facilitates its incorporation into quantitative imaging protocols. This can be useful in future applications, including quantitative perfusion and relaxometry mapping. Lastly, enhancing resilience to $B_{1}/B_{0}$ inhomogeneities could enable broader clinical adoption.

## Figures and Tables

**Table 1:** Characteristics and suppression performance of the different pulses used in the MB-OVS module.

			AM SINC X			SINC X				
Parameters	No OVS	RF-0	AM SINC 5	AM SINC 8	AM SINC 11	SINC 5	SINC 10	SINC 20	SINC 30	SINC 40
Duration [ms]			3.84	3.84	3.84	1.92	3.84	7.68	11.52	15.36
TBW [-]			5	8	11	5	10	20	30	40
SAR [W/kg]	0.09	0.007	0.81	1.26	1.60	1.40	1.46	1.35	1.27	1.16
Residual Signal [%]	100	110.8	32.69	23.72	24.14	27.34	24.95	24.02	24.08	22.21
Gr [μT/m]			203	326	448	407	407	407	407	407
$B_{1}^{+}$ [μT]			8.44	12.8	16.4	15.4	14.7	15.6	15.1	15.4

## Data Availability

The data underlying this research will be made available to interested researchers upon a request sent to the corresponding author.

## Supplementary Materials

The following supporting information can be downloaded at: https://www.mdpi.com/article/10.3390/bioengineering13030286/s1, Video S1: CINE images of a representative slice (#5) with 5-fold SMS factor and 2-fold further in-plane acceleration without MB-OVS module; Video S2: CINE images of a representative slice (#5) with 5-fold SMS factor and 2-fold further in-plane acceleration with MB-OVS module.

## Abbreviations

The following abbreviations are used in this manuscript:

SMS	Simultaneous Multi-Slice
OVS	Outer Volume Suppression
RF	Radio Frequency
MB	Multi-Band
SAR	Specific Absorption Rate
PE	Phase-encoding
TBW	Time-Bandwidth product
spGRE	Spoiled Gradient Echo
ROI	Regions Of Interest
ACS	Auto Calibration Signal
LLR	Locally Low Rank
PD-DL	Physics-Driven Deep Learning
SIIM	Signal Intensity Informed Multi-coil
LV	Left Ventricle
SSDU	Self-Supervised Learning via Data Undersampling
