# Supplementary figures and images for: Efficient Interleaved Multi-Band Outer Volume Suppression for Highly Accelerated Simultaneous Multi-Slice Imaging of the Heart
Source: Bioengineering (Basel). 2026 Feb 28;13(3):286. doi: 10.3390/bioengineering13030286 (PMC13024669; doi:10.3390/bioengineering13030286)

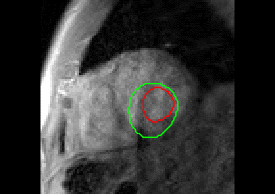

Supplement: Supplementary file 1 [file bioengineering-13-00286-s001.zip › Supplementary/Video_S1.gif]

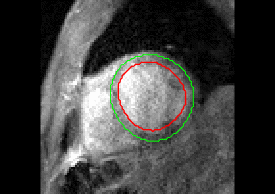

Supplement: Supplementary file 1 [file bioengineering-13-00286-s001.zip › Supplementary/Video_S2.gif]
